# Genome-wide analysis of aberrant methylation in human breast cancer cells using methyl-DNA immunoprecipitation combined with high-throughput sequencing

**DOI:** 10.1186/1471-2164-11-137

**Published:** 2010-02-25

**Authors:** Yoshinao Ruike, Yukako Imanaka, Fumiaki Sato, Kazuharu Shimizu, Gozoh Tsujimoto

**Affiliations:** 1 Department of Genomic Drug Discovery Science, Graduate School of Pharmaceutical Sciences, Kyoto University, 46-20 Yoshida Shimoadachi-cho Sakyo-ku, Kyoto 606-8501, Japan; 2 Department of Nanobio Drug Discovery, Graduate School of Pharmaceutical Sciences, Kyoto University, 46-20 Yoshida Shimoadachi-cho Sakyo-ku, Kyoto 606-8501, Japan

## Abstract

**Background:**

Cancer cells undergo massive alterations to their DNA methylation patterns that result in aberrant gene expression and malignant phenotypes. However, the mechanisms that underlie methylome changes are not well understood nor is the genomic distribution of DNA methylation changes well characterized.

**Results:**

Here, we performed methylated DNA immunoprecipitation combined with high-throughput sequencing (MeDIP-seq) to obtain whole-genome DNA methylation profiles for eight human breast cancer cell (BCC) lines and for normal human mammary epithelial cells (HMEC). The MeDIP-seq analysis generated non-biased DNA methylation maps by covering almost the entire genome with sufficient depth and resolution. The most prominent feature of the BCC lines compared to HMEC was a massively reduced methylation level particularly in CpG-poor regions. While hypomethylation did not appear to be associated with particular genomic features, hypermethylation preferentially occurred at CpG-rich gene-related regions independently of the distance from transcription start sites. We also investigated methylome alterations during epithelial-to-mesenchymal transition (EMT) in MCF7 cells. EMT induction was associated with specific alterations to the methylation patterns of gene-related CpG-rich regions, although overall methylation levels were not significantly altered. Moreover, approximately 40% of the epithelial cell-specific methylation patterns in gene-related regions were altered to those typical of mesenchymal cells, suggesting a cell-type specific regulation of DNA methylation.

**Conclusions:**

This study provides the most comprehensive analysis to date of the methylome of human mammary cell lines and has produced novel insights into the mechanisms of methylome alteration during tumorigenesis and the interdependence between DNA methylome alterations and morphological changes.

## Background

DNA methylation is an indispensable epigenetic modification of mammalian genomes. In mammals, it occurs predominantly at CpG dinucleotides which are sparsely distributed through the genome except at short genomic regions called CpG islands (CGIs) [1]. The state of CpG methylation regulates and stabilizes chromatin structure, and possibly regulates accessibility of these DNA regions to the transcription machinery [2]. DNA methylation is essential to diverse processes such as development, X-inactivation, and imprinting [3-5]. Alterations to the normal patterns of DNA methylation are linked to many human diseases, such as cancer [6]. Many studies have explored the aberrant patterns of DNA methylation in cancers, as they might possibly be of value as cancer cell markers, markers of tumor prognosis, predictors of response to chemotherapy, and therapeutic targets [7-10]. Human tumors have been shown to undergo a massive loss of DNA methylation, but also to become hypermethylated at certain gene promoters [11]. However, the entire genomic distribution of aberrant methylations and the molecular mechanisms underlying the methylome alterations in cancers remain unclear, mainly due to the limitations of existing techniques for analyzing DNA methylation at specific sequences [12]. For example, the conventional strategies using methylation-sensitive restriction enzymes require high-molecular-weight DNA and are limited by the sequence context of the chosen enzyme.

Recently an important technical advance for analyzing DNA methylation was made by using immunoprecipitation with an antibody against 5-methylcytosine to enrich methylated DNA fragments [8]. This methyl-DNA immunoprecipitation (MeDIP)-based approach enables the rapid identification of multiple CpG sites universally, and it can be combined with gene-by-gene PCR detection and with several promoter, CGI and tiling microarrays [13-16]. However, predefined CGIs cover only 7.4% of all CpGs in the human genome and the entire human genome is not yet represented in any microarray. Analysis of DNA methylation has also been advanced recently by the application of high-throughput DNA sequencing technology that allows robust, quantitative, and cost-effective functional genomic strategies. MeDIP in conjunction with high-throughput sequence (MeDIP-seq) provides a genome-wide mapping technique that has successfully been used to profile the global DNA methylation patterns of mature human spermatozoa genome [17]. Bisulphite sequencing has also been combined with high-throughput sequence (BS-seq) to describe the 120 Mb Arabidopsis DNA methylome [18,19]. In addition, BS-seq was recently applied to the human DNA methylome [20]. Unfortunately, it still remains too hard work to apply BS-seq on a multiple comparative analysis of methylomes in mammalian genomes.

In this study, we used MeDIP-seq to investigate the whole-genome distribution of aberrant DNA methylation in eight breast cancer cells (BCC) lines and compared these with the methylation patterns of normal human mammary epithelial cells (HMEC). Furthermore, to investigate the mechanisms of methylome alteration and determine the effects of such changes on the morphology of BCC lines, we identified alterations to the methylation profile that occurred during the epithelial-to-mesenchymal transition (EMT) in MCF7 cells treated with TGFβ and TNFα. Using this experimental approach, we obtained novel insights in to the molecular and genetic mechanisms of methylome alterations in BCC lines and their functional association with cancer phenotype.

## Results

### High-throughput sequencing analysis of MeDIP DNA

We profiled the genome-wide DNA methylation status of normal and cancerous mammary cells by first generating MeDIP-enriched DNA libraries. Real-time quantitative PCR was used for several genomic regions that included known methylated or unmethylated promoters to confirm that MeDIP specifically enriched methylated DNA and efficiently removed unmethylated DNA (Additional file 1a). Immunoprecipitated fractions were subjected to high-throughput sequencing using an Illumina Genome Analyzer to obtain comprehensive methylation maps. The influence of genomic amplifications and deletions in the BCC lines was investigated by high-throughput sequencing of input DNA fragments from each samples, with the exception of MDAMB453 and MRKnu1 which yielded an insufficient number of reads. We obtained 97 million uniquely mapped singleton reads and 11 million paired-end reads for the MeDIP samples and 26 million singleton reads for the input samples with high quality read placements against the human genome (Maq quality > 10). The mapping of input reads allowed the efficient detection of genomic amplifications, including known regions of amplification (for example, 17q23 and 20q13 in MCF7) [21,22]. We excluded these regions from the following analyses as they might result in failure to identify regions with hypermethylation. Overall, 87% of genomic CpGs all over the genome were covered by any sample with 12 times depth, the average of each sample's maximum depth (Additional file 1b). Thus, our data sets successfully provided non-biased genome-wide information. We observed that some pairs in the paired-end reads had identical outer coordinates. As this should happen rarely, we assumed this to be due to PCR biases introduced by the whole-genome amplification after immunoprecipitation. We therefore removed any duplicates from each data set to improve the overall reproducibility in the following analyses.

To confirm the reproducibility of the analyses, we performed two replicates of the MeDIP-seq experiments with HMEC. The numbers of reads overlapping each CGI and 100 kb genomic segment were highly correlated between the replicates (r = 0.92 and 0.99, respectively) (Additional file 1c). To assess whether the MeDIP-seq analyses correctly identified methylated regions, we compared MCF7 MeDIP-seq results with previously reported MCF7 promoter methylome data generated by the MeDIP-chip technique (deposited in the public Gene Expression Omnibus database: GSM263125 [13]). For each probe region, we counted the number of MeDIP-enriched reads and subtracted the number of input reads. Overall, regions detected by MeDIP-seq were found to have higher methylation levels in MeDIP-chip results., indicating accurate detection of methylated regions by MeDIP-seq (Additional file 1d). We also observed considerable hypomethylation at some repeat sequences, such as human satellite II (HSATII, Additional file 1e), known to be primary targets of hypomethylation in many cancers [23].

A number of studies have reported that CGIs are hypermethylated in many cancers [2,12,24]; we therefore investigated the differential methylation status of CGIs. Compared to HMEC, we found a more than four-fold increase in methylation levels in CGIs in many BCC lines (Additional file 2a). The known hypermethylation status of many CGIs, for example WT1 [25] and HOXA5 [26], was confirmed by the MeDIP-seq data sets (Additional file 2b). We also identified other CGIs highly methylated in BCC lines and confirmed their methylation levels using bisulfite sequencing (Additional file 2c, d). Additionally, we perfomed bisulfite sequencing on randomly chosen regionsto confirm our MeDIP-seq results (data not shown). We observed a strong correlation between the MeDIP-seq data and bisulfite sequencing data, confirming the reliablity of the MeDIP-seq data (Additional file 2e).

### Genomic distribution of aberrant DNA methylation in BCC lines

We thought it would be useful to obtain overview methylation maps, albeit at reduced resolution, to understand the pattern of methylation at the genome-wide level. We divided the entire genome into 100 kb segments and counted the number of reads mapped within each segment. To compare methylation levels within each sample, the number of reads were normalized against the total number of reads; and the inferred number of reads per 10 million total reads was calculated for each sample. The normalized number of reads in 100 kb regions was clearly correlated to the number of CpGs in the HMEC MeDIP sample (Additional file 3a), whereas the input sample showed nearly constant values irrespective of the number of CpGs. Compared to HMEC, BCC lines showed a lower correlation between the number of reads and CpGs, suggesting broadly altered methylation levels in BCC lines.

**Figure 1:**
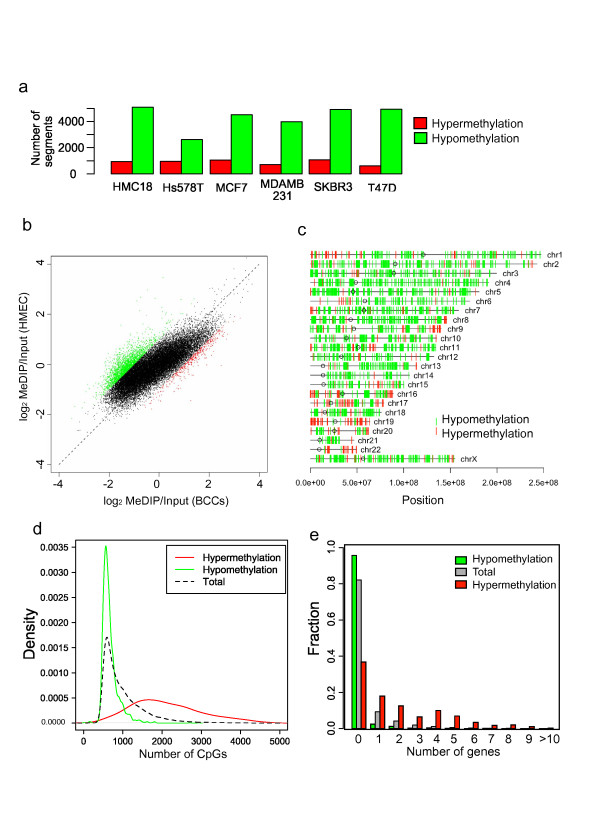
**BCCs undergo massive overall loss of methylation**. (a) The number of differentially methylated 100 kb segments were counted for each cell lines. (b) Log scaled scatter plot of the MeDIP/Input ratio in each 100 kb segments for HMEC and BCCs (for BCCs the average ratio is shown). The red and green dots show hyper- and hypomethylation respectively. The dashed line shows a diagonal line. (c) Genome-wide distribution of hyper- or hypomethylated regions. Red bars indicate hypermethylated regions and green bars indicate hypomethylated regions. Circles indicate centromeres. (d) The distribution of the number of CpGs within hyper- or hypomethylated regions. (e) The distribution of the number of genes within hyper- or hypomethylated regions.

The number of MeDIP-reads were divided by the input and used for pairwise comparisons among the samples, in order to classify each segment as hyper-, hypo-, and not-differentially methylated groups. We defined hypermethylation and hypomethylation in BCC lines as a two-fold increase and decrease, respectively, in the normalized tag frequency compared to HMEC. In all BCC lines, hypomethylation occurred 3 to 5 times more frequently than hypermethylation (Figure 1a). Hierarchical clustering showed distinct methylation patterns of HMEC, compared to BCC lines (Additional file 3b and 3c). Compared to HMEC, 8.2% of the segments in BCC lines showed on average a two-fold decrease in methylation levels (Figure 1b). Next, we investigated the distribution of hypomethylated and hypermethylated positions across the entire genome. Hypomethylated regions in BCC lines were distributed throughout entire genome, while hypermethylated regions were clustered at specific loci (1.3% of all segments), including 16p13.3, 19p13.3, and 9q34.3 (Figure 1c). The number of CpGs within the hypermethylated and hypomethylated segments was highly biased toward a lower or higher frequency of CpGs, respectively (Figure 1d). Hypermethylation was also positively correlated with the number of genes within the segment region (Figure 1e). All BCC lines showed significantly higher methylation levels in gene-rich regions. In contrast, HMEC showed a lower association between methylation levels and gene frequency (Additional file 3d), suggesting that aberrant methylation preferentially targets genes. We also performed these analyses using smaller window sizes (~10 kb) and obtained consistent results (data not shown).

### Target position of aberrant methylation in BCC lines

**Figure 2:**
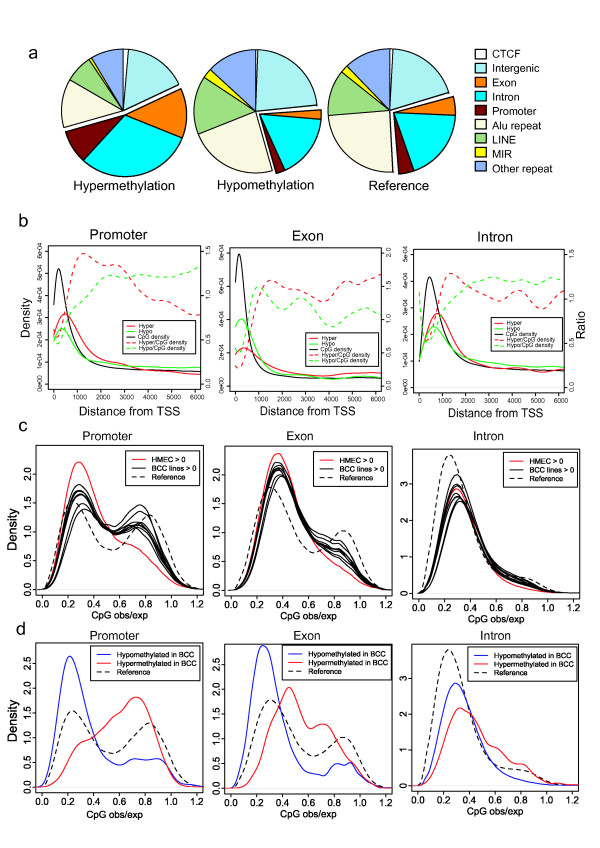
**Hypermethylations are associated with CpG-rich and gene-related regions**. (a) Pie chart representing the proportions of each genomic features of hyper- or hypomethylated CpGs. The repeats are not included when considering subsequent features. Promoters are defined as 10 kb regions from transcriptional start sites annotated in RefSeq database. (b) Distribution of the distance from transcription start sites to differentially methylated sites. CpG density is shown as a black line. Dotted lines show the ratio of hyper- or hypomethylated CpGs to CpG density. (c) Distribution of CpG_o/e _ratios surrounding CpGs covered in each MeDIP samples. (d) Distribution of CpG_o/e _ratios surrounding hyper- or hypomethylated CpGs in BCCs.

We sought to determine whether the positions of aberrantly methylated CpGs were related to genomic features. We defined promoter regions as the 10 kb upstream region of all transcription start sites deposited in the RefSeq database. Exons and introns were also defined by this database. The repetitive sequences were excluded. As ratios could not be determined when methylated DNA fragments were not detected by MeDIP-seq in one sample, we evaluated methylation status by a qualitative criterion in this analysis; we defined hypermethylation as the region covered by all BCCs but not by HMEC, and hypomethylation as the region covered by HMEC but not by any BCCs. We found that the majority of hypermethylated CpGs were related to gene promoters, exons and introns (Figure 2a). By contrast, hypomethylated CpGs did not show significant enrichment of any defined genomic feature. Hypermethylated CpGs were also found within the hypomethylated 100 kb segments described above and were preferentially found at gene-related features (Additional file 3e). We then investigated the distance between aberrantly methylated gene-related positions and transcription start sites (TSS). The CpGs around TSS were classified into promoters, exons and introns, and then we calculated their distances to TSS. Hyper- and hypomethylated CpGs tended to be concentrated in regions proximal to TSS, suggesting that these regions are important for the regulation of gene expression (Figure 2b). Since the density of CpGs was highest at regions proximal to TSS, the relative frequencies of hyper- and hypomethylations at distal regions were higher than proximal regions, when considering the ratio with the CpG content. We also analyzed the distribution of the distance from transcription start sites to hyper- and hypomethylated CpGs in CpG islands (Additional file 4a) and found that relative frequencies of hypermethylations at distal regions were higher than proximal regions.

To examine the effect of methylations both proximal and distal to TSS on gene expression, we performed a correlation analysis between methylation patterns and gene expression. The gene expression profiles of six BCC lines (Hs578T, MCF7, MDAMB231, MDAMB453, SKBR3, T47D) were obtained from a public database (Gene Expression Omnibus: GSE12777) [27]. Regions around TSS (+/- 10 kb) were divided into 1 kb segments and the number of reads within each segment was counted for each sample. The fraction of genes with expressions positively correlated to methylation patterns was higher at distal regions (Additional file 4b). On the other hand, the fraction of genes with expressions negatively correlated was higher at proximal regions, although the fraction of negatively correlated genes were lower than positively correlated genes at any distance from TSS.

### Aberrant methylations at CpG-rich regions in BCCs

As described above, hypermethylation was associated with CGIs. However, the hypermethylation observed at gene-related regions was not limited to predefined CGIs (Additional file 5a): 53% of hypermethylated CpGs in BCC lines belonged to non-CGI regions. Our observations therefore challenge the current presumption that hypermethylation is restricted to CGIs and proximal promoters. We investigated the characteristics of hypermethylated CpGs across the entire genome by counting the number of neighboring CpGs within the 500 bp regions surrounding hypermethylated sites and calculated the CpG observed/expected ratio (CpG_o/e_) [1] for each CpG.

To screen for association between CpG_o/e _and aberrant methylation targets, we examined the CpG_o/e _distribution across all CpGs covered by each MeDIP sample. The CpG_o/e _ratio distribution of CpGs that belonged to promoter regions was divided into two distinct classes at the CpG_o/e _ratio 0.6, which is also the criterion for CGIs [1,28]. The promoter CpGs covered in HMEC were considerably biased towards a low ( < 0.6) CpG_o/e _ratio (Figure 2c and Additional file 5b). On the other hand, CpGs with a high (>0.6) CpG_o/e _ratio were highly covered by all BCC lines. We also found that hypermethylation of a CpG-rich region was more likely to be shared by BCC lines, compared to that in a CpG-poor region (Additional file 5c). By contrast, hypomethylation in a CpG-poor region was more likely to be shared by BCC lines, compared to that in CpG-rich region. We then defined the hypermethylated CpGs that were covered in all BCC lines but not in HMEC, and hypomethylated CpGs that were covered in HMEC but not in all BCC lines. Hypermethylation was mainly observed in CpGs with a high CpG_o/e _ratio, and hypomethylation was highly biased towards CpGs with a low CpG_o/e _ratio (Figure 2d). Hypermethylation was also observed at exons and introns in high CpG_o/e _regions.

### EMT-induced methylome alteration

**Figure 3:**
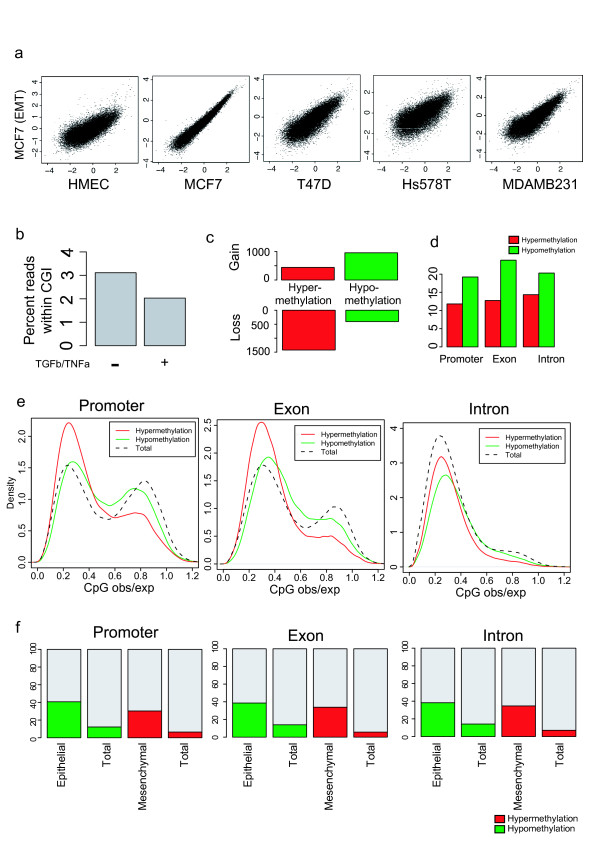
**EMT-induced methylome alterations in MCF7**. (a) Log scaled scatter plot displaying the association between the MeDIP/Input ratio of 100 kb segments in EMT-induced MCF7 and each cell lines. (b) Bar plot displaying the percentage of reads covering CGIs in EMT-induced or control MCF7. (c) The number of hyper- or hypomethylated CGIs gained or lost through EMT-induction in MCF7. (d) Bar plot displaying the percentage of hyper- or hypomethylated CpGs within each gene-related regions. (e) Distribution of CpG_o/e _surrounding CpGs, hyper- or hypomethylated through EMT. (f) Bar plot displaying the percentage of hyper- or hypomethylated CpGs within cell-type specifically methylated regions.

According to the epithelial-mesenchymal transition (EMT) hypothesis, BCCs undergo phenotype alterations during the sequential in vivo progression of cancer from atypical hyperproliferation to metastatic disease, subtended or not by genetic changes [29,30]. Although altered methylations of some gene promoters have been reported to be one of the principal causes of EMT [31,32], the relationship between the epithelial-mesenchymal phenotype of breast cancers and the genome-wide methylation profile remains unclear. Here, we screened for genome-wide methylome alterations during EMT induction in MCF7. Treatment with TGFβ and TNFα efficiently altered the normal epithelial phenotype of MCF7 cells to a mesenchymal phenotype. Pairwise comparisons of normalized methylation levels in 100 kb segments revealed that EMT had little effect on the global distribution of DNA methylation (Figure 3a). However, compared to normal MCF7 cells, the percentage of reads covering CGIs was reduced following induced EMT (Figure 3b). EMT induction was associated with a massive loss of hypermethylated CGIs and gain of hypomethylated CGIs (Figure 3c). Furthermore, EMT induction more frequently resulted in hypomethylation rather than hypermethylation at gene-related CpGs (Figure 3d), and EMT-induced hypomethylation was predominant especially at CpG-rich regions (Figure 3e).

We also investigated whether EMT-induced methylome alterations in CpG-rich regions were associated with epithelial- or mesenchymal-specific patterns of methylation. Based on the phenotypic classification of cell lines into epithelial (HMEC, MCF7 and T47D) and mesenchymal (HMC18, Hs578T and MDAMB231) [29], epithelial- or mesenchymal-specific methylation patterns were determined. Approximately 40% of epithelial-specific methylation sites were hypomethylated following EMT-induction, while only 10% of total sites were hypomethylated (Figure 3f). In addition, approximately 40% of mesenchymal-specific methylation sites were hypermethylated following EMT-induction, while only ~10% of total sites were hypermethylated. These cell-type specific methylome alterations were found in all gene-related regions. Although these changes are partial, the results suggest that EMT-induction affects the morphological phenotype of the cells through methylome alterations within CpG-rich regions.

## Discussion

This study provides the first comprehensive, detailed map of DNA methylation patterns in human mammary cell lines. Methylated DNA fragments were detected using a highly sensitive method involving enrichment by MeDIP, and high-throughput sequencing enabled the non-biased mapping of aberrant methylation sites across the genomes of BCC lines. We examined 10 different cell populations in total, including EMT-induced MCF7 cells, to investigate the association between DNA methylome alterations and changes in cellular morphological phenotypes. Our data sets covered almost the entire genome with sufficient depth to identify differentially methylated regions, thereby providing high resolution and reproducibility, and proved that MeDIP-seq is a cost-effective approach for comparative analyses of the mammalian DNA methylome. Although many researchers have sought to describe DNA methylome alterations in cancers, to our knowledge this is the first methylome study that effectively encompasses the entire genome and is not limited to specific sequences. This study has thus provided important new insights into the biological implications of DNA methylation.

Firstly, the global methylation map provides an indication of the intricate relationship between the methylome and transcriptome. Most cancer methylome studies have assumed that functionally important DNA methylation is restricted to promoters and that most DNA methylation changes in cancer occur in CGIs, and they ignore all other regions. In our analysis, although hypermethylation at predefined CGIs was a remarkable feature of the BCC lines, we also found that many non-CGI regions were broadly hypermethylated. Hypermethylation occurred not only at proximal promoters but also at exons and introns, including regions distal from the TSS. Since DNA methylation interrupts the binding of transcription factors to their response elements [2,11], changes in methylation at distal regions may affect the expression of a gene. Since proximal promoters or CGI methylation are not always correlated with gene expression [13,33], our comprehensive methylation maps will help us to better understand methylation-dependent transcriptional regulation.

Secondly, the comprehensive methylation maps help us to understand how methylation is maintained and how it is disrupted in BCC lines. We found that the number of reads was positively correlated with the number of CpGs within 100 kb segments and this relationship was especially clear in HMEC. Although HMEC showed relatively low methylation levels in gene-related CpG-rich regions (corresponding to less than 8% of whole-genome CpGs), overall, methylation of each CpG seemed to be maintained at a nearly constant level. The most prominent feature of aberrant methylation was the massive overall hypomethylation and simultaneous hypermethylation at CpG-rich regions. While these observations are consistent with previous reports [11], our results provide the most detailed map of aberrant methylation in BCC lines. We found that hypomethylation in BCC lines was biased towards CpG-poor regions, and analysis of genomic features indicated there was no preference for repeat sequences but included all gene-related regions equally. Furthermore, hypermethylations of CpG-rich gene-related regions was present in BCC lines although within extremely hypomethylated 100 kb segments. These results support the idea that there are at least two distinct maintenance mechanisms that produce the aberrant methylation patterns in CpG-poor regions and CpG-rich regions. One mechanism maintains CpGs at a constant methylation level, which is downregulated in BCC lines. Another mechanism maintains gene-related CpG-rich regions at a specific methylation level, which is generally upregulated in BCC lines.

Finally, the comparative analysis of methylation maps of MCF7 cells with or without EMT induction provided an increased understanding of how cells undergo DNA methylome alterations. Analysis of 100 kb segments revealed almost unchanged overall methylation levels throughout EMT, while hypomethylation was observed at many CGI sites. With respect to the two distinct mechanisms of methylome alteration described above, EMT had little effect on the maintenance of the overall methylation level, but had a considerable effect on cell-type specific methylation. On the other hand, EMT induction altered cell-type specific gene-related methylation such that hypomethylation was predominantly observed at CpG-rich regions. The idea that there are cell-type and gene specific mechanisms for regulation of methylation patterns within CpG-rich gene-related regions received further support from the following observations. The methylation status within CpG-rich regions were likely to be shared by BCC lines, and CpGs within CpG-rich repetitive sequences such as HSATII were extensively hypomethylated in BCC lines. Considering that the methylation status participates in determining the morphological phenotypes of the cells [7,28,29], our results suggested an interdependence between cell-type and gene specific regulation of DNA methylations and morphological changes during EMT.

## Conclusions

We performed a comprehensive methylation profiling of human mammary cell lines using the MeDIP-seq analysis, revealing aberrant methylation patterns in BCC lines and EMT-induced alteration of the methylome. Methylome alteration in BCC lines had two principal characteristics: a downregulated overall level of CpG methylation; and cell-type specific regulation of DNA methylation at gene-related CpG-rich regions. Our results provide important insights into the mechanisms of methylome alterations during tumor development.

## Methods

### Cell lines

HMEC (CC-2551, Lonza) was cultured using the medium supplied by MEGM Bullet Kit (CC-3150, Lonza) at 37°C and 5% CO_2_. MCF7, MDAMB231, SKBR3, and T47D were kindly gifted by Dr. Masakazu Toi (professor of Kyoto University Hospital Breast Surgery Department) and cultured in RPMI 1640 medium (Invitrogen) containing 10% FBS. Hs578T (86082104, European Collection of Cell Culture) and MDAMB453 (RCB1192, RIKEN BioResource Center) were cultured in DMEM containing 10% FBS. HMC-1-8 and MRK-nu-1 (JCRB0166 and JCRB0628 respectively, Health Science Research Resources Bank) were cultured in RPMI 1640 medium (Invitrogen) containing 10% FBS.

### Bisulfite-modified DNA sequencing

We prepared genomic DNA from cultured cells using the Gentra Puregene Cell kit (Qiagen). Approximately 2 μg of DNA was bisulfite-treated with the EpiTect Bisufite kit according to the manufacturer's protocol (Qiagen). Amplification across the entire bisulfite converted genome was performed by the EpiTect Whole Bisufitome kit according to the manufacturer's protocol (Qiagen). Nested PCR was performed on bisulfite-modified DNA using the following primers; IRX1firstF: GTTTTTGTATATTTGGTGGA, IRX1firstR: CAACTATCTAACAACCTATC, IRX1nestedF: TTTTTGGGTGAAGAGAAAGT, IRX1nestedR: CCCTTTTTAACAAAAACAAC, PAX7firstF: GGGAGTTTTATTGGAGGAAT, PAX7firstR: ACTCCCTCCCTCTTCTCCAC, PAX7nestedF: GAGAAGATGAGAAATAGGGT, PAX7nestedR: TCCACACCAACTTTCACAAC.

### Methyl-DNA immunoprecipitation

Before carrying out MeDIP, we sonicated genomic DNA to produce random fragments ranging in size from 200 to 600 bp. We used 4 mg of fragmented DNA for a standard MeDIP assay as described [14]. Briefly, following denaturation at 95°C for 10 min, immunoprecipitation was carried at 4°C for 2 hr using 10 μg of monoclonal antibody against 5-methylcytidine (315-80541, Diagenode) in a final volume of 500 μl IP buffer (10 mM sodium phosphate (pH 7.0), 140 mM NaCl, 0.05% Triton X-100). We incubated the mixture with 40 μl of Dynabeads and M-280 sheep antibody to mouse IgG (Dynal Biotech) for 12 hr at 4°C and washed it seven times with 700 μl of IP buffer. We then treated the beads with proteinase K for 4 hr at 50°C and recovered the methylated DNA by phenol-chloroform extraction followed by ethanol precipitation.

### Illumina Genome Analyzer sequencing

We performed second strand synthesis of MeDIP-enriched single-strand DNA fragments. Samples containing ~200 ng of DNA and 500 ng of random primer at a final volume of 57.9 μl were incubated at 70°C for 10 min, followed by gradual cooling for 40 min. Two ml of 2.5 mM each dNTPs, 20 μl of 5× second strand buffer (100 mM Tris-HCl (pH 7.5), 500 mM potasium chloride, 25 mM magnesium chloride, 50 mM ammonium sulphate, and 250 mg/ml bovine serum albumin), 10 μl of 100 mM dithiothreitol, 3 μl of 5 mM beta-NAD+, 0.5 μl (5 U) of *E. coli *DNA ligase (TaKaRa Bio), and 6.6 μl (25 U) of *E. coli *DNA polymerase I (TaKaRa Bio) were then added to the sample (100 μl final volume). The reaction was performed at 14°C for 12 hr. Next, we purified double-stranded DNA fragments using the PCR purification kit (Qiagen). The end-repair of DNA fragments, addition of an adenine to the 3' ends of DNA fragments, adaptor ligation, and PCR amplification by Illumina paired-end PCR primers were performed as described previously [14]. We performed gel electrophoresis and excised bands from the gel to produce libraries with insert sizes of 250~350 bp, and quantified these libraries using the Quant-iT PicoGreen dsDNA Reagent and Kits (Invitrogen). We then prepared flowcells with 8 pM DNA using the manufacture's recommended protocol and sequenced for 36 cycles on an Illumina Genome Analyzer II. Obtained images were analyzed and base-called using GA pipeline software version 1.3 with default settings provided by Illumina. MeDIP-seq data from this study have been submitted to DDBJ Read Archive database http://trace.ddbj.nig.ac.jp/dra/index_e.shtml under accession number "DRA000030".

### Mapping reads

We downloaded the human genome sequence and mapping information (Mar. 2006, hg18) from the University of California Santa Cruz Genome Bioinformatics Site http://genome.ucsc.edu. The reads were mapped onto the human genome reference sequence using the high-performance alignment software 'maq' version 0.7.1 http://maq.sf.net[17]. The reads with maq quality less than 10 were removed from further analysis. We considered each singleton read as a 250 bp block extended from the single read's mapping position on its strand, representing an entry for MeDIP-enriched DNA fragments.

### EMT-induction

MCF7 cells were plated at 3 × 10^4 ^cells/ml in 12 well plates and incubated for 24 hr. Cells were then treated with 2 ng/ml of TGFβ and 10 ng/ml of TNFα for five days [34]. EMT-induction was confirmed by morphological alterations and the detection of downregulated epithelial marker expression and upregulated mesenchymal marker expression using reverse transcription PCR.

## Abbreviations

DNA: deoxyribonucleic acid; MeDIP: methyl-DNA immunoprecipitation; MeDIP-seq: MeDIP combined with high-throughput sequencing; BCC: breast cancer cell; HMEC: human mammary epitherial cell; EMT: epithelial-mesenchymal transition; CGI: CpG island; BS-seq: bisulfite sequencing; HSATII: human satelite II; CpG_o/e_: CpG observed/expected ratio; TGFβ: tumor growth factor beta 1; TNFα: tumor necrosis factor (TNF superfamily, member 2); WT1: Wilms tumor 1; HOXA5: homeobox A5; IRX1: iroquois homeobox 1; PAX7: paired box 7.

## Authors' contributions

All authors conceived the experiments. YR and YI performed the experiments and YR analyzed data. YR and GT co-wrote the paper. All authors discussed the results and commented on the manuscript. All authors read and approved the final manusucript.

## Supplementary Material

Additional file 1 **Supplemental Figure 1**. A figure showing effieient detection of methylation by MeDIP-seq. Click here for file

Additional file 2 **Supplemental Figure 2**. A figure showing aberrant methylation patterns of CGIs in BCCs. Click here for file

Additional file 3 **Supplemental Figure 3**. A figure showing the correlation between the normalized number of reads and the number of CpGs and genes within 100 kb genomic segments. Click here for file

Additional file 4 **Supplemental Figure 4**. A figure showing aberrant methylation at the region distal from TSS. Click here for file

Additional file 5 **Supplemental Figure 5**. A figure showing differently regulated methylation of CpG-poor and CpG-rich regions. Click here for file

## References

[B1] Gardiner-Garden M Frommer M CpG islands in vertebrate genomes J Mol Biol 1987 196 261 282 10.1016/0022-2836(87)90689-9 3656447

[B2] Clark SJ Melki J DNA methylation and gene silencing in cancer: which is the guilty party? Oncogene 2002 21 5380 5387 10.1038/sj.onc.1205598 12154400

[B3] Li E Bestor TH Jaenisch R Targeted mutation of the DNA methyltransferase gene results in embryonic lethality Cell 1992 69 915 926 10.1016/0092-8674(92)90611-F 1606615

[B4] Li E Beard C Jaenisch R Role for DNA methylation in genomic imprinting Nature 1993 366 362 365 10.1038/366362a0 8247133

[B5] Migeon BR Concerning the role of X-inactivation and DNA methylation in fragile X syndrome Am J Med Genet 1992 43 182 187 10.1002/ajmg.1320430145 1605203

[B6] Feinberg AP Tycko B The history of cancer epigenetics Nat Rev Cancer 2004 4 143 153 10.1038/nrc1279 14732866

[B7] Hu M Yao J Cai L Bachman KE Brûle F van den Velculescu V Polyak K Distinct epigenetic changes in the stromal cells of breast cancers Nat Genet 2005 37 899 905 10.1038/ng1596 16007089

[B8] Weber M Davies JJ Wittig D Oakeley EJ Haase M Lam WL Schübeler D Chromosome-wide and promoter-specific analyses identify sites of differential DNA methylation in normal and transformed human cells Nat Genet 2005 37 853 862 10.1038/ng1598 16007088

[B9] Irizarry RA Ladd-Acosta C Wen B Wu Z Montano C Onyango P Cui H Gabo K Rongione M Webster M Ji H Potash JB Sabunciyan S Feinberg AP The human colon cancer methylome shows similar hypo- and hypermethylation at conserved tissue-specific CpG island shores Nat Genet 2009 41 178 186 10.1038/ng.298 19151715 PMC2729128

[B10] Weber M Hellmann I Stadler MB Ramos L Pääbo S Rebhan M Schübeler D Distribution, silencing potential and evolutionary impact of promoter DNA methylation in the human genome Nat Genet 2007 39 457 466 10.1038/ng1990 17334365

[B11] Herman JG Baylin SB Gene silencing in cancer in association with promoter hypermethylation N Engl J Med 2003 349 2042 2054 10.1056/NEJMra023075 14627790

[B12] Esteller M Cancer epigenomics: DNA methylomes and histone-modification maps Nat Rev Genet 2007 8 286 298 10.1038/nrg2005 17339880

[B13] Komashko VM Acevedo LG Squazzo SL Iyengar SS Rabinovich A O'Geen H Green R Farnham PJ Using ChIP-chip technology to reveal common principles of transcriptional repression in normal and cancer cells Genome Res 2008 18 521 532 10.1101/gr.074609.107 18347325 PMC2279240

[B14] Pomraning KR Smith KM Freitag M Genome-wide high throughput analysis of DNA methylation in eukaryotes Methods 2009 47 142 150 10.1016/j.ymeth.2008.09.022 18950712

[B15] Pelizzola M Koga Y Urban AE Krauthammer M Weissman S Halaban R Molinaro AM MEDME: an experimental and analytical methodology for the estimation of DNA methylation levels based on microarray derived MeDIP-enrichment Genome Res 2008 18 1652 1659 10.1101/gr.080721.108 18765822 PMC2556264

[B16] Irizarry RA Ladd-Acosta C Carvalho B Wu H Brandenburg SA Jeddeloh JA Wen B Feinberg AP Comprehensive high-throughput arrays for relative methylation (CHARM) Genome Res 2008 18 780 790 10.1101/gr.7301508 18316654 PMC2336799

[B17] Down TA Rakyan VK Turner DJ Flicek P Li H Kulesha E Gräf S Johnson N Herrero J Tomazou EM Thorne NP Bäckdahl L Herberth M Howe KL Jackson DK Miretti MM Marioni JC Briney E Hubbard TJ Drubin R Tavaré S Beck S A Bayesian deconvolution strategy for immunoprecipitation-based DNA methylome analysis Nat Biotechnol 2008 26 779 785 10.1038/nbt1414 18612301 PMC2644410

[B18] Lister R O'Malley RC Tonti-Filippini J Gregory BD Berry CC Millar AH Ecker JR Highly integrated single-base resolution maps of the epigenome in Arabidopsis Cell 2008 133 523 536 10.1016/j.cell.2008.03.029 18423832 PMC2723732

[B19] Cokus SJ Feng S Zhang X Chen Z Merriman B Haudenschild CD Pradhan S Nelson SF Pellegrini M Jacobsen SE Shotgun bisulphite sequencing of the Arabidopsis genome reveals DNA methylation patterning Nature 2008 452 215 219 10.1038/nature06745 18278030 PMC2377394

[B20] Lister R Pelizzola M Dowen RH Hawkins RD Hon G Tonti-Filippini J Nery JR Lee L Ye Z Ngo QM Edsall L Antosiewicz-Bourget J Stewart R Ruotti V Millar AH Thomson JA Ren B Ecker JR Human DNA methylomes at base resolution show widewpread epigenomic differences Nature 2009 462 315 322 10.1038/nature08514 19829295 PMC2857523

[B21] Wu GJ Sinclair CS Paape J Ingle JN Roche PC James CD Couch FJ 17q23 amplifications in breast cancer involve the PAT1, RAD51C, PS6K, and SIGma1B genes Cancer Res 2000 60 5371 5375 11034073

[B22] Bärlund M Monni O Weaver JD Kauraniemi P Sauter G Heiskanen M Kallioniemi OP Kallioniemi A Cloning of BCAS3 (17q23) and BCAS4 (20q13) genes that undergo amplification, overexpression, and fusion in breast cancer Genes Ghromosomes Cancer 2002 35 311 317 10.1002/gcc.10121 12378525

[B23] Jackson K Yu MC Arakawa K Fiala E Youn B Fiegl H Müller-Holzner E Widschwendter M Ehrlich M DNA hypomethylation is prevalent even in low-grade breast cancers Cancer Biol Ther 2004 3 1225 1231 15539937 10.4161/cbt.3.12.1222

[B24] Tommasi S Karm DL Wu X Yen Y Pfeifer GP Methylation of homeobox genes is a frequent and early epigenetic event in breast cancer Breast Cancer Res 2009 11 R14 10.1186/bcr2233 19250546 PMC2687719

[B25] Laux DE Curran EM Welshons WV Lubahn DB Huang TH Hypermethylation of the Wilms' tumor suppressor gene CpG island in human breast carcinomas Breast Cancer Res 1999 56 35 43 10.1023/A:1006222803788 10517341

[B26] Novak P Jensen T Oshiro MM Wozniak RJ Nouzova M Watts GS Klimecki WT Kim C Futscher BW Epigenetic inactivation of the HOXA gene cluster in breast cancer Cancer Res 2006 66 10664 10670 10.1158/0008-5472.CAN-06-2761 17090521

[B27] Hoeflich KP O'Brien C Boyd Z Cavet G Guerrero S Jung K Januario T Savage H Punnoose E Truong T Zhou W Berry L Murray L Amler L Belvin M Friedman LS Lackner MR In vivo antitumor activity of MEK and phosphatidylinositol 3-kinase inhibitors in basal-like breast cancer models Clin Cancer Res 2009 15 4649 64 10.1158/1078-0432.CCR-09-0317 19567590

[B28] Saxonov S Berg P Brutlag DL A genome-wide analysis of CpG dinucleotides in the human genome distinguishes two distinct classes of promoters Proc Natl Acad Sci USA 2006 103 1412 1417 10.1073/pnas.0510310103 16432200 PMC1345710

[B29] Lacroix M Leclercq G Relevance of breast cancer cell lines as models for breast tumours: an update Breast Cancer Res Treat 2004 83 249 289 10.1023/B:BREA.0000014042.54925.cc 14758095

[B30] Lombaerts M van Wezel T Philippo K Dierssen JW Zimmerman RM Oosting J van Eijk R Eilers PH Water B van de Cornelisse CJ Cleton-Jansen AM E-cadherin transcriptional downregulation by promoter methylation but not mutation is related to epithelial-to-mesenchymal transition in breast cancer cell lines Br J Cancer 2006 94 661 671 16495925 10.1038/sj.bjc.6602996 PMC2361216

[B31] Cheng AS Culhane AC Chan MW Venkataramu CR Ehrich M Nasir A Rodriguez BA Liu J Yan PS Quackenbush J Nephew KP Yeatman TJ Huang TH Epithelial progeny of estrogen-exposed breast progenitor cells display a cancer-like methylome Cancer Res 2008 68 1786 1796 10.1158/0008-5472.CAN-07-5547 18339859 PMC4172329

[B32] Dumont N Wilson MB Crawford YG Reynolds PA Sigaroudinia M Tlsty TD Sustained induction of epithelial to mesenchymal transition activates DNA methylation of genes silenced in basal-like breast cancers Proc Natl Acad Sci USA 2008 105 14867 14872 10.1073/pnas.0807146105 18806226 PMC2567459

[B33] Jones PA The DNA methylation paradox Trends Genet 1999 15 34 37 10.1016/S0168-9525(98)01636-9 10087932

[B34] Burk U Schubert J Wellner U Schmalhofer O Vincan E Spaderna S Brabletz T A reciprocal repression between ZEB1 and members of the miR-200 family promotes EMT and invasion in cancer cells EMBO Rep 2008 9 582 589 10.1038/embor.2008.74 18483486 PMC2396950

