# Supplementary material for: Genome-wide analysis of aberrant methylation in human breast cancer cells using methyl-DNA immunoprecipitation combined with high-throughput sequencing
Source: BMC Genomics. 2010 Feb 25;11:137. doi: 10.1186/1471-2164-11-137 (PMC2838848; doi:10.1186/1471-2164-11-137)

Supplemental Figure 1. Confirming efficient detection of methylation by MeDIP-seq

(a) Real time quantitative PCR analysis for known methylated (AX1) and unmethylated (GAPDH) promoters. Positive control DNA fragments processed by in vitro methylation reactions and unprocessed negative control DNA fragments were mixed in input samples and enriched by MeDIP. Unmethylated DNA fragments were efficiently removed in MeDIP samples, and detected in input samples. (b) Pie chart representing the fraction of CpGs covered with each depth. (c) Scatter plot showing the correlation between two replicated MeDIP-seq experiments. HMEC methylations for each 100kb segments (left panel) and CGIs (right panel) are shown. (d) Box-and-whisker plot showing correlation between MeDIP-seq data and MeDIP-chip data (GSM263125). Boxes, bars and whiskers represent interquartile range, median and 1.5 times extended interquartile range of MeDIP-chip signal distributions, respectively. Overall, regions detected by MeDIP-seq were found to have higher methylation levels in MeDIP-chip results. (e) Representative methylation patterns of HSATII.

Supplementary Figure 1

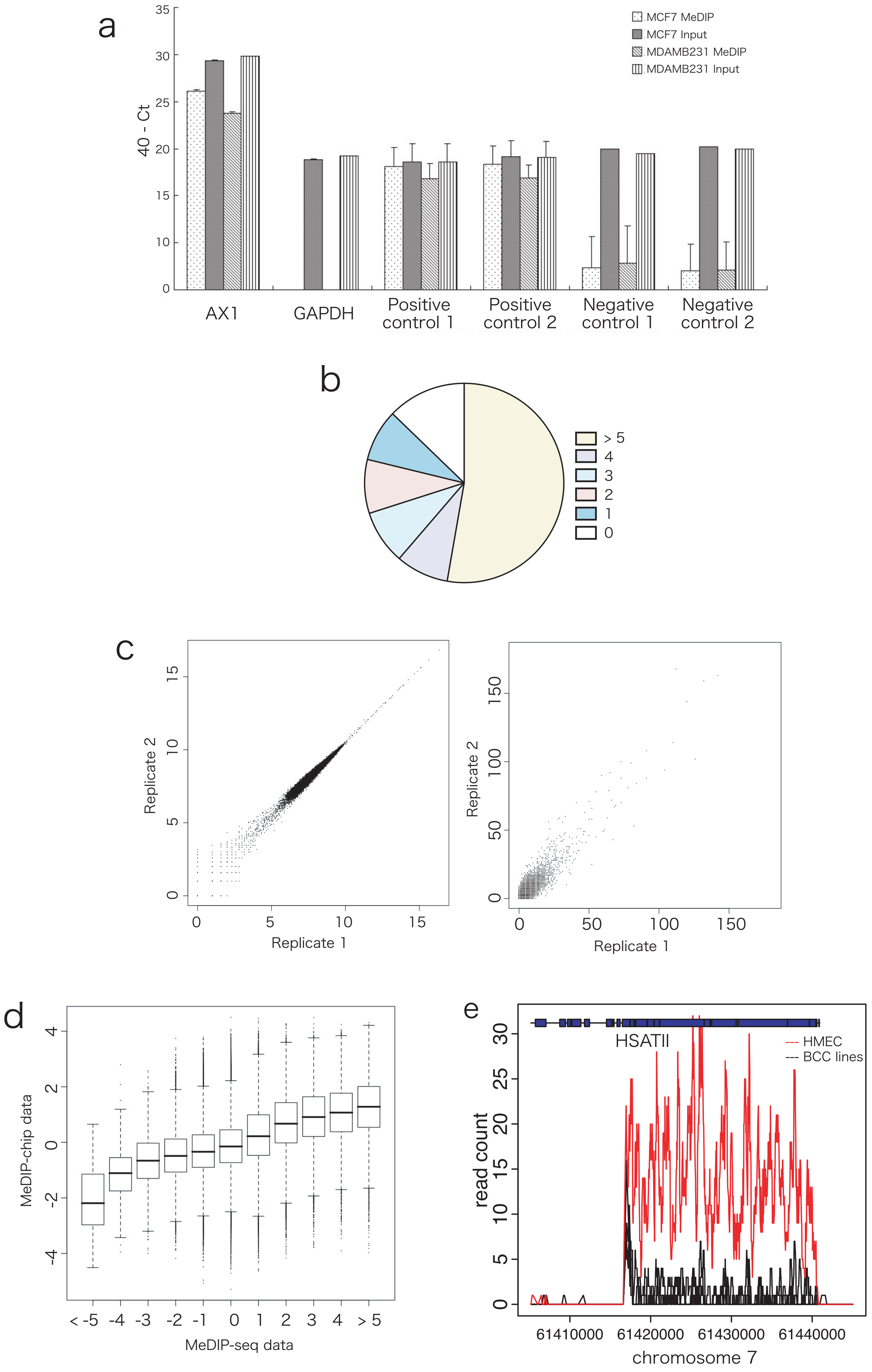

Supplement: Additional file 1 — Supplemental Figure 1. A figure showing effieient detection of methylation by MeDIP-seq. [file 1471-2164-11-137-S1.PDF]
