# Supplementary material for: Genome-wide analysis of aberrant methylation in human breast cancer cells using methyl-DNA immunoprecipitation combined with high-throughput sequencing
Source: BMC Genomics. 2010 Feb 25;11:137. doi: 10.1186/1471-2164-11-137 (PMC2838848; doi:10.1186/1471-2164-11-137)

Supplemental Figure 2. Aberrant methylation patterns of CGIs in BCCs

(a) Number of hyper- or hypomethylated CGIs in BCCs. (b) Methylation patterns of WT1 (left panel) and Hox5 (right panel) promoter regions in each cell lines. (c) Methylation patterns of IRX1 (left panel) and PAX7 (right panel) promoter regions. The regions amplified for bisulfite-modified sequence analysis were indicated as red bars. (d) Bisulfite-modified sequence analysis for regions shown in (c). The percentage of methylated CpGs in 4 clones of each cell lines were shown on the right side.

Supplementary Figure 2

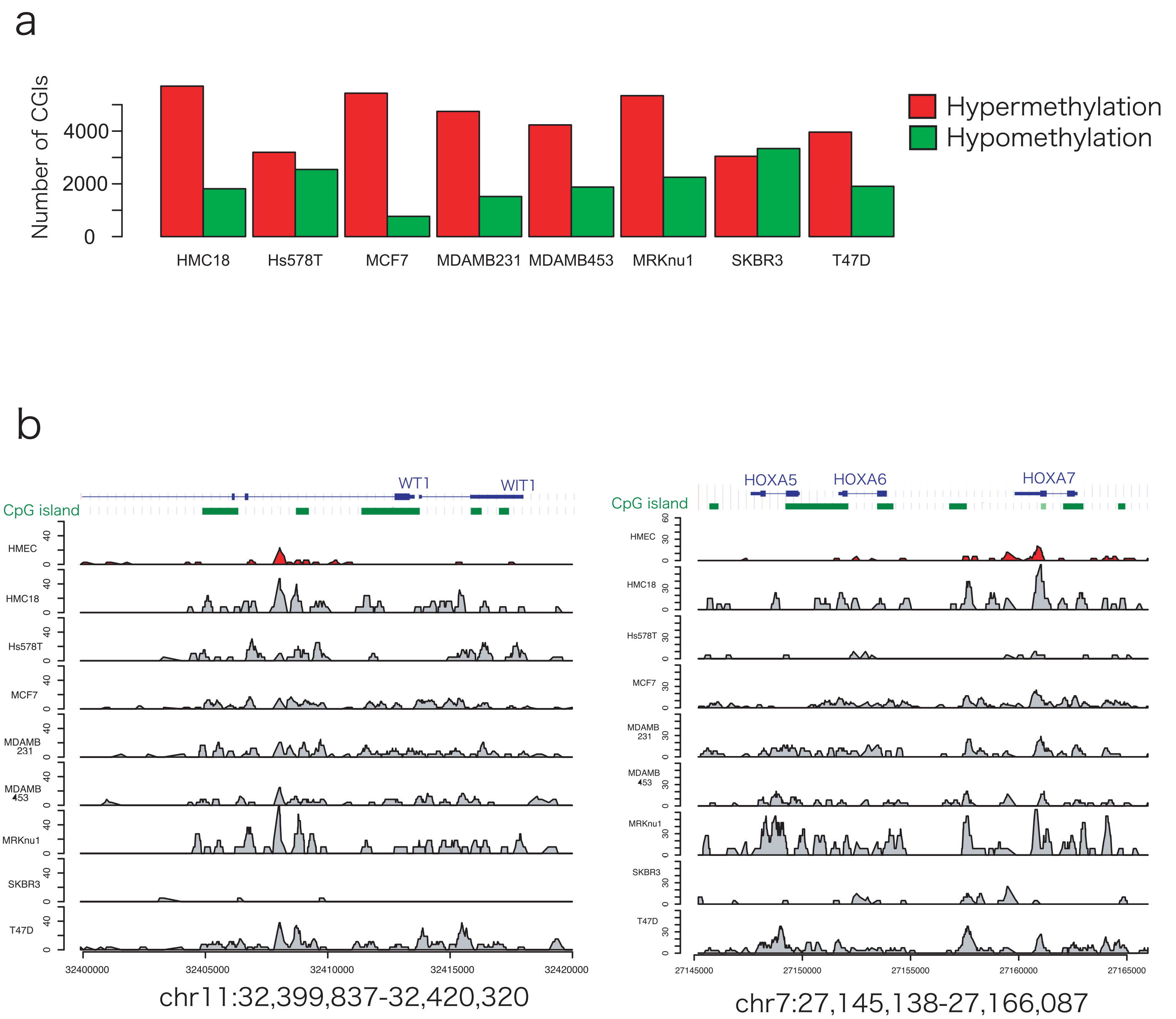

Supplemental Figure 2 (continue)

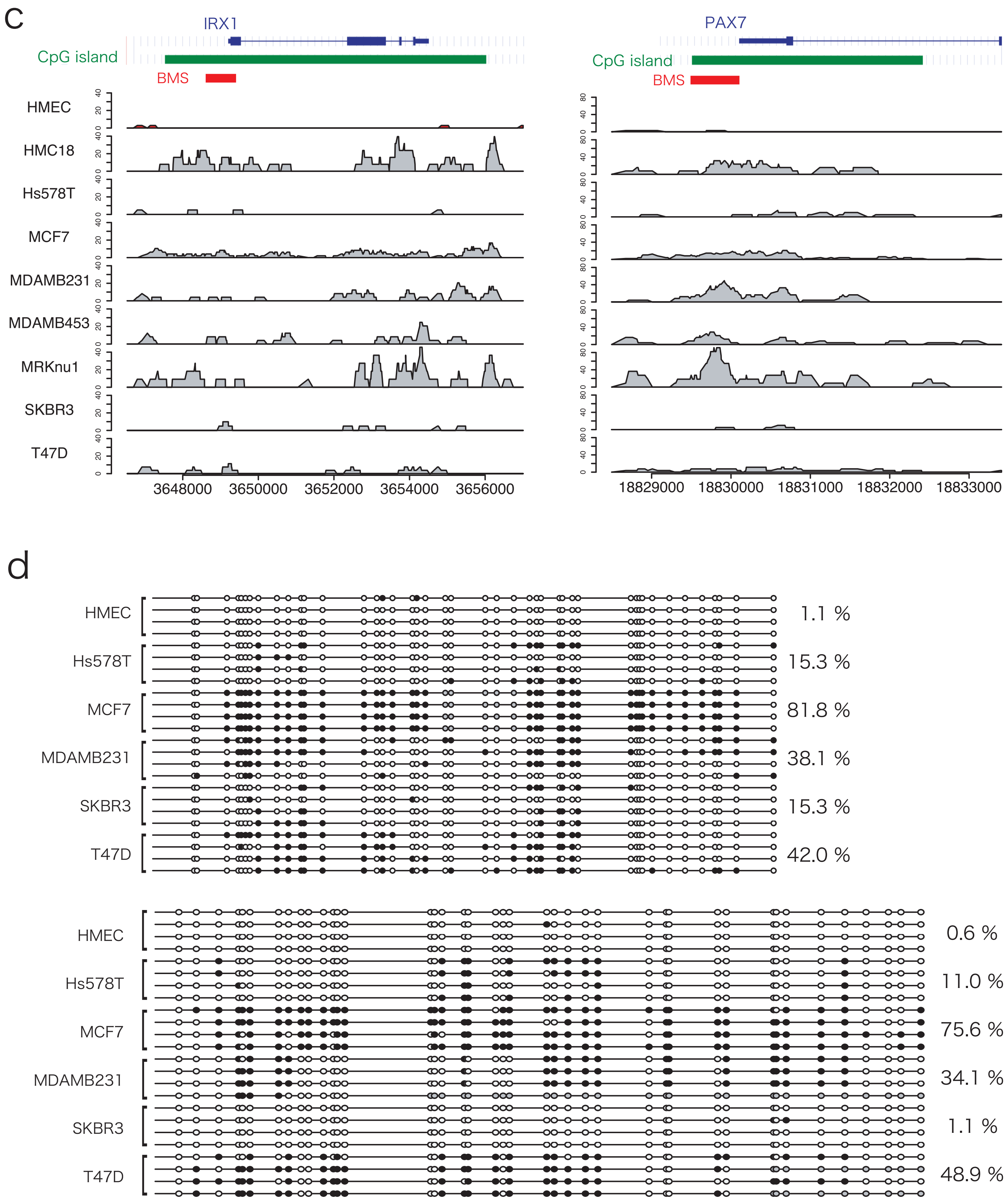

Supplement: Additional file 2 — Supplemental Figure 2. A figure showing aberrant methylation patterns of CGIs in BCCs. [file 1471-2164-11-137-S2.PDF]
