# Supplementary material for: Genome-wide analysis of aberrant methylation in human breast cancer cells using methyl-DNA immunoprecipitation combined with high-throughput sequencing
Source: BMC Genomics. 2010 Feb 25;11:137. doi: 10.1186/1471-2164-11-137 (PMC2838848; doi:10.1186/1471-2164-11-137)

Supplemental Figure 3. The noramalized number of reads correlate to the number of CpGs and genes within 100kb genomic segments

(a) Log scaled scatter plot showing the correlation between the number of CpGs within 100kb segments and the normalized number of reads per 10 million reads in each samples. Red lines represent the estimated linear model using regression analysis. The values of slopes and intercepts are shown in supplemental table. (b) Pairwise correlation analysis among the methylomes of HMEC and BCC lines. (c) Hierarchical clustering analysis based on the pairwise correlation of methylation patterns. (d) Box-and-whisker plot representing the distribution of MeDIP/Input ratios for each levels of gene density. (e) Pie chart representing the proportions of each genomic features of hyper- or hypomethylated CpGs within hyper- or hypomethylated 100kb segments.

Supplemental Figure 3

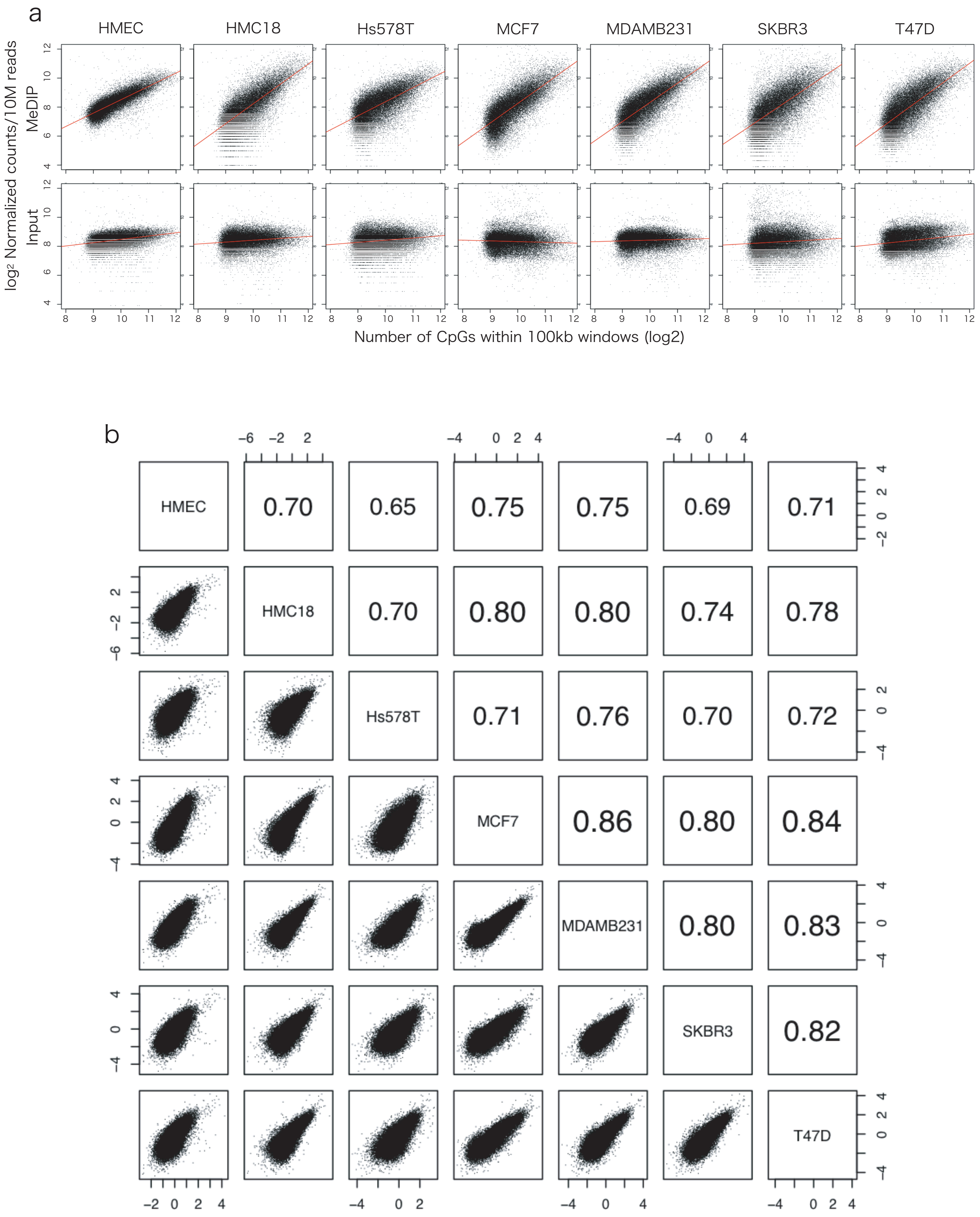

Supplemental Figure 3 (continued)

c

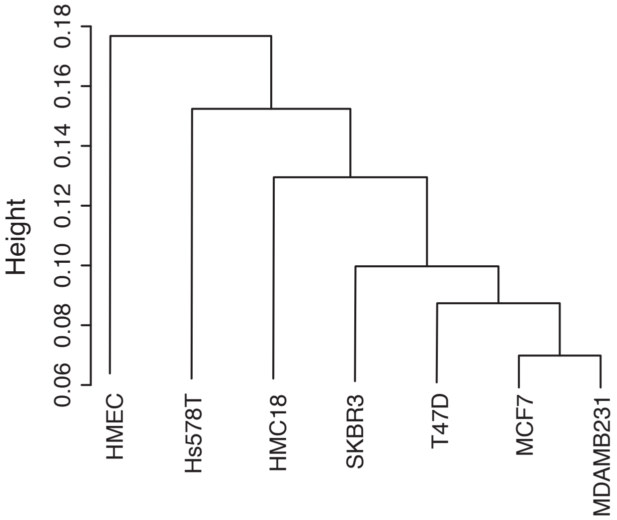

d

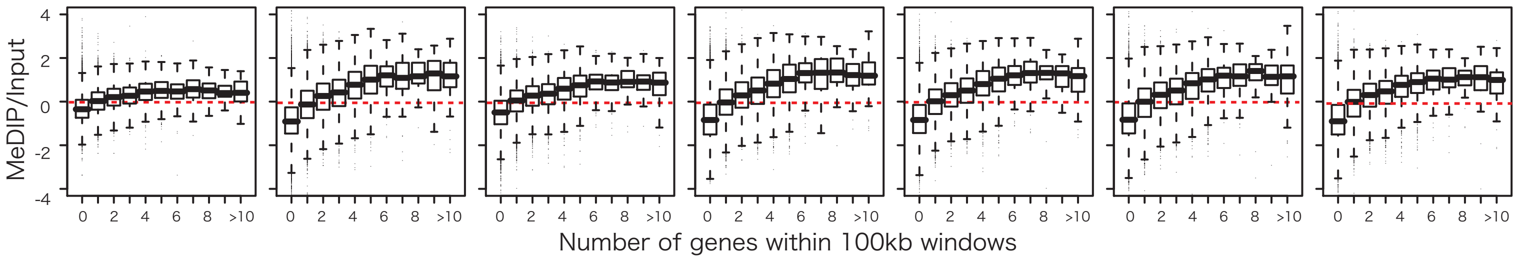

e

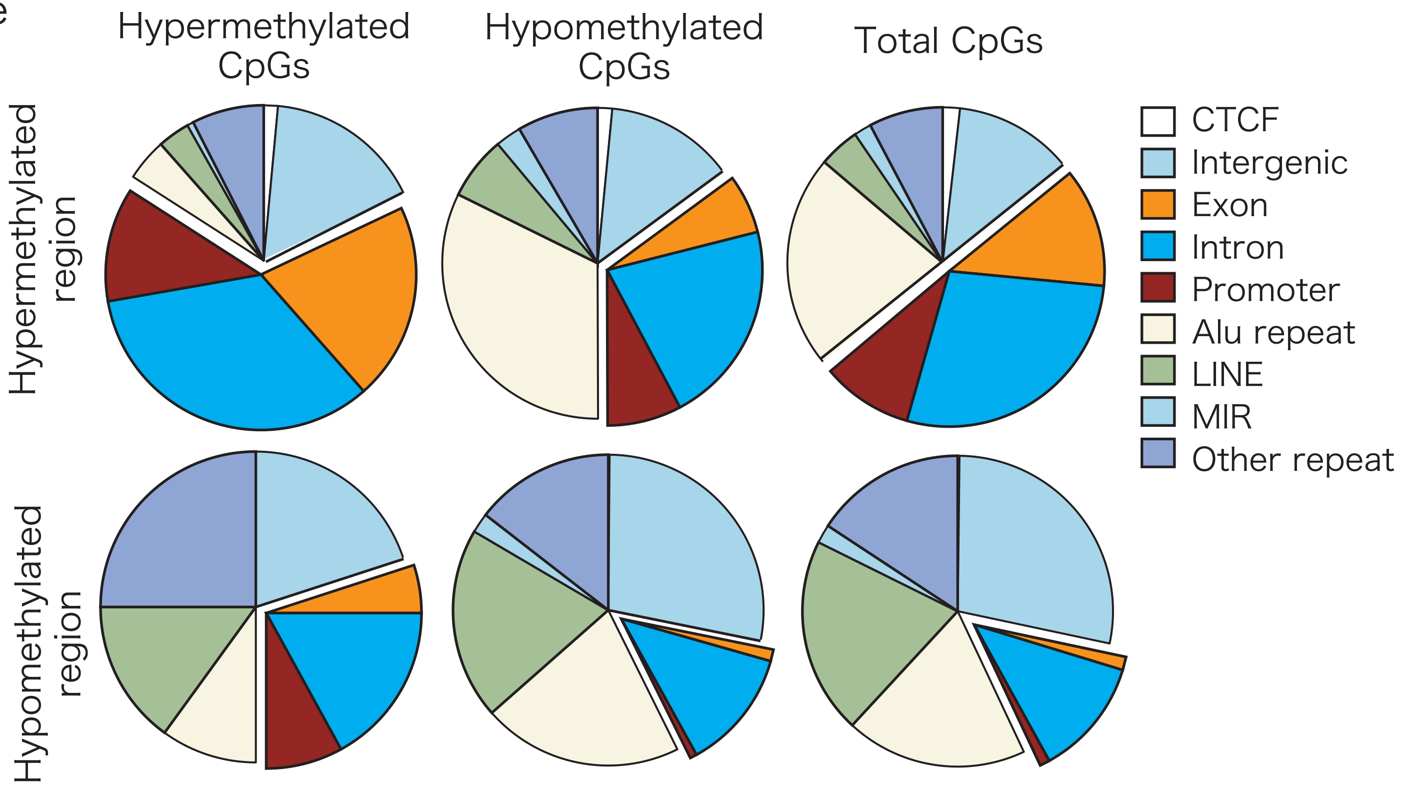

Supplement: Additional file 3 — Supplemental Figure 3. A figure showing the correlation between the normalized number of reads and the number of CpGs and genes within 100 kb genomic segments. [file 1471-2164-11-137-S3.PDF]
