# Supplementary material for: Genome-wide analysis of aberrant methylation in human breast cancer cells using methyl-DNA immunoprecipitation combined with high-throughput sequencing
Source: BMC Genomics. 2010 Feb 25;11:137. doi: 10.1186/1471-2164-11-137 (PMC2838848; doi:10.1186/1471-2164-11-137)

Supplemental Figure 4. Aberrant methylation at the region distal from TSS.

(a) Distribution of the distance from transcription start sites to differentially methylated sites in CpG islands. CpG density is shown as a black line. Dotted lines show the ratio of hyper- or hypomethylated CpGs to CpG density. (b) Correlation analysis between gene expression and methylation patterns of regions around TSS. The fraction of genes positively or negatively correlated (ppc: pearson's correlation constants > 0.6 or < -0.6, respectively) was plotted.

Supplemental Figure 4

a

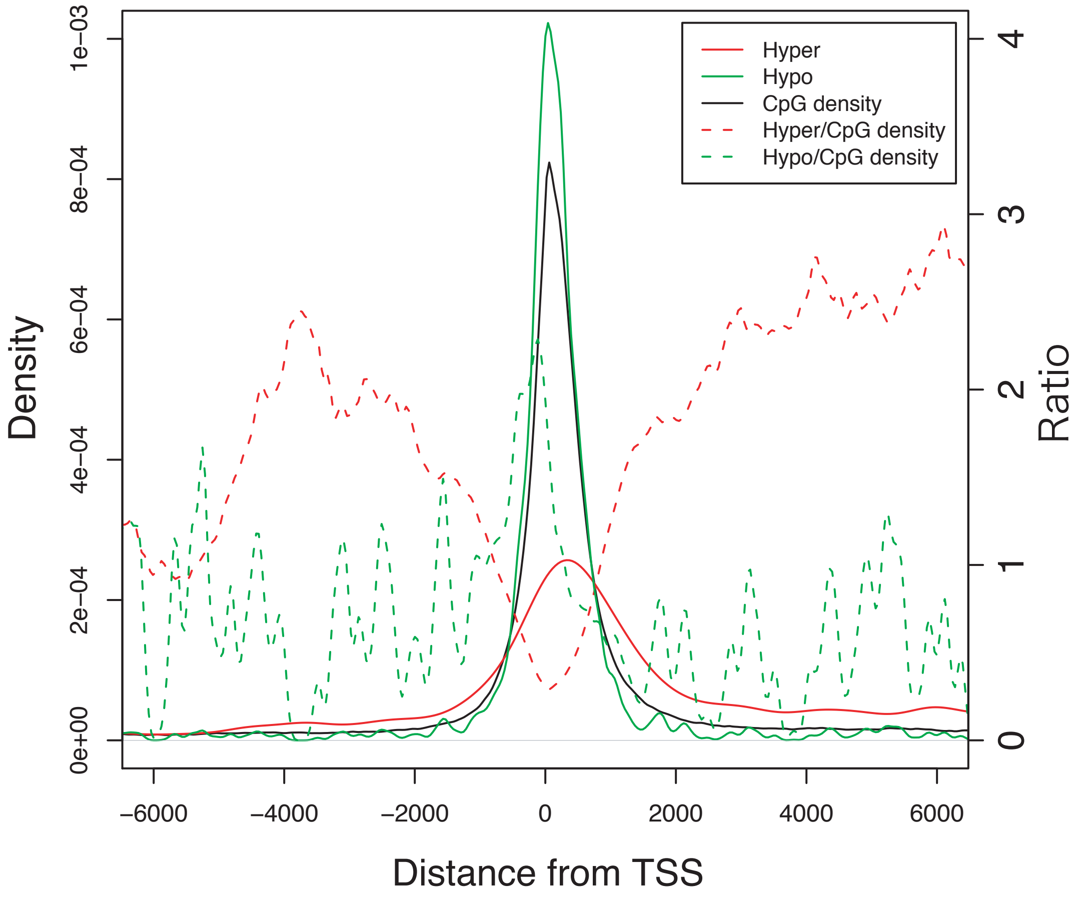

b

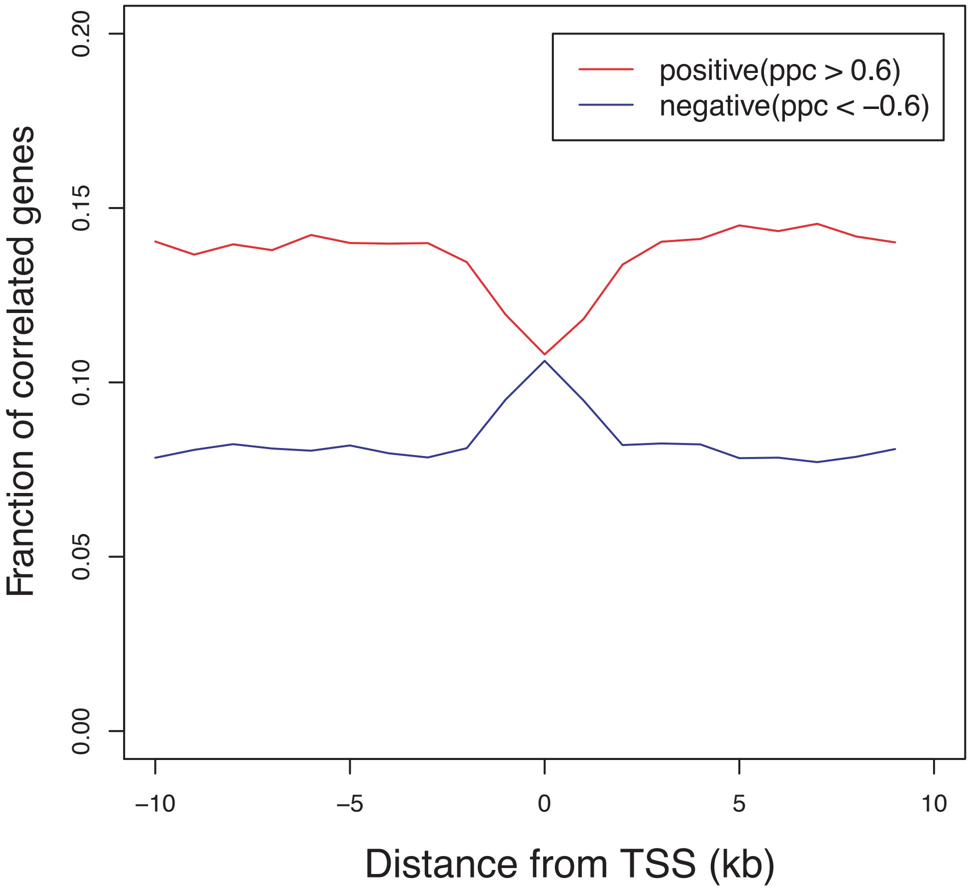

Supplement: Additional file 4 — Supplemental Figure 4. A figure showing aberrant methylation at the region distal from TSS. [file 1471-2164-11-137-S4.PDF]
