# Supplementary material for: Genome-wide analysis of aberrant methylation in human breast cancer cells using methyl-DNA immunoprecipitation combined with high-throughput sequencing
Source: BMC Genomics. 2010 Feb 25;11:137. doi: 10.1186/1471-2164-11-137 (PMC2838848; doi:10.1186/1471-2164-11-137)

Supplemental Figure 5. Differently regulated methylation of CpG-poor and CpG-rich regions.

(a) The number of hyper- (left panel) or hypomethylated (right panel) CpGs within each genomic features. Non-CGI CpGs were represented by white bars and CpGs within CGIs were represented by black bars. (b) The percentage of CpG-poor and CpG-rich promoter, exon and intron regions covered in each cell line (c) For each CpG covered (left panel) or not covered (right panel) by HMEC MeDIP sample, the fraction of CpGs shared by 0~6 BCCs was examined.

Supplementary Figure 5

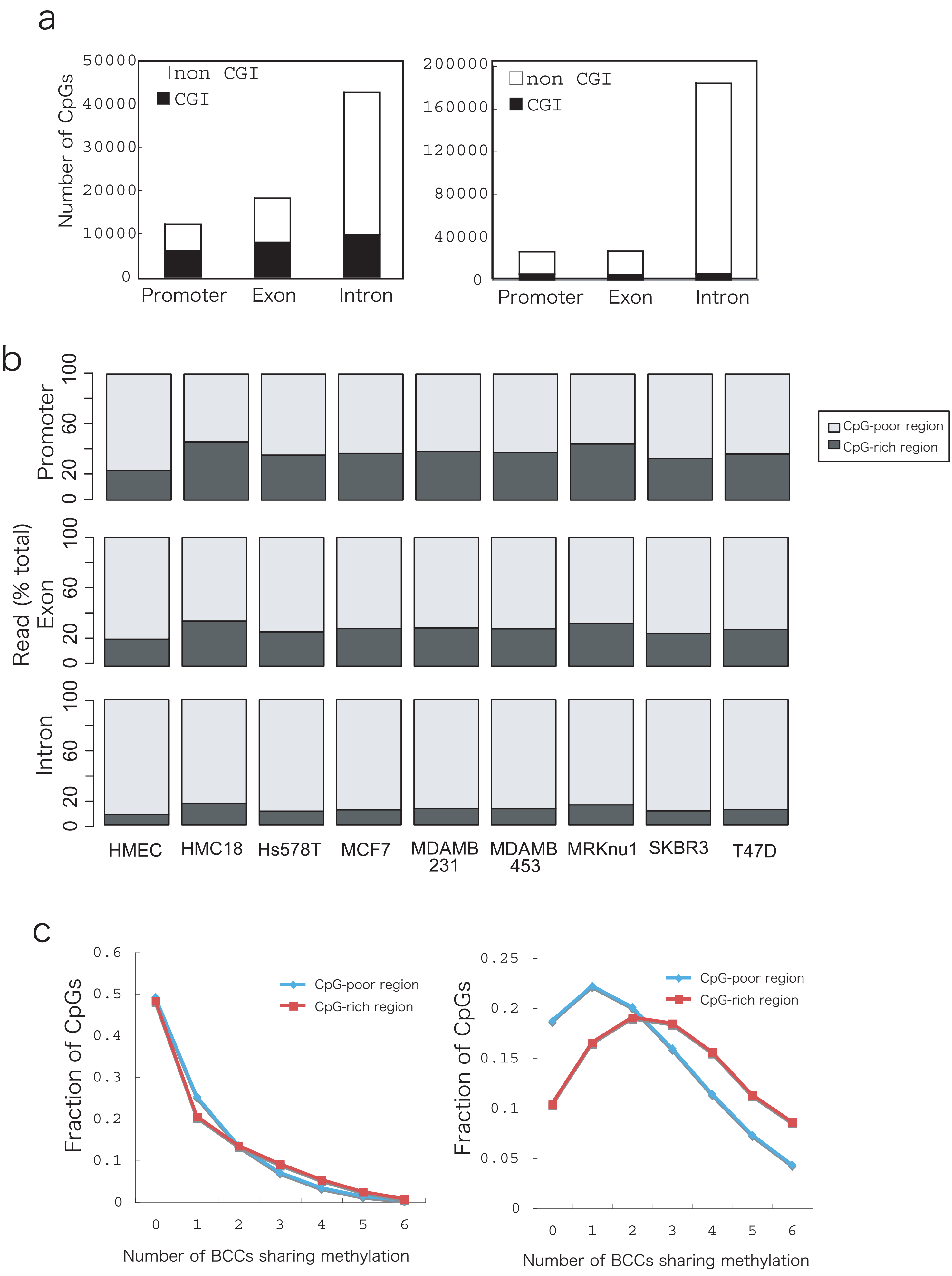

Supplement: Additional file 5 — Supplemental Figure 5. A figure showing differently regulated methylation of CpG-poor and CpG-rich regions. [file 1471-2164-11-137-S5.PDF]
